# The Local Problem

**Published:** 1951-04

**Authors:** William Hughes

**Affiliations:** Area Geriatrician, Bristol


### GERIATRICS.

### III : THE LOCAL PROBLEM

BY

WILLIAM HUGHES, M.D., M.R.C.P
*Area Geriatrician, Bristol*

During the past year I have been occupied partly in making a survey of the aged sick in the Bristol clinical area and partly in trying to develop a scheme to deal with the local geriatric problem. The population of the Bristol clinical area is about 650,000 of whom 13.5 per cent., or 87,750, can be assumed to be over the age of sixty years. Rowntree's (1947) figure of 1.16 per cent. of old people actually in Public Assistance Institutions in May, 1946, would amount to 1,018 for the area; but Sheldon (1948) suggests that 2 per cent. need institutional care giving a figure of 1,755. If we apply the figure of 2.5 per cent. given by Dr. Andrews for Cornwall we should expect to find 2,200 needing hospital or hostel accommodation. Actually the number needing institutional care is much higher than this.

I found a total population of 1,735 occupying hospital beds as shown in the following table:

MAIN CONCENTRATIONS OF AGED SICK IN BRISTOL CLINICAL AREA

| Hospital or Institution | Population |
|---|---|
| Stapleton .. .. .. | 526 |
| Bristol Mental Hospital .. | 392 |
| Snowdon Road .. .. | 296 |
| 100 Fishponds Road .. | 120 |
| Frenchay .. .. .. | 39 |
| Chipping Sodbury* .. | 108 |
| Thornbury .. .. .. | 96 |
| Axbridge .. .. .. | 58 |
| General Hospitals .. .. | 100† |
| TOTAL .. .. | 1,735 |

* These beds have now been handed back to the Local Authority.
† Approximate figure.

In addition there are 850 ambulant old people in local authority and voluntary hostels in Bristol and an unidentified number in similar institutions outside Bristol city which I was unable to visit. A figure of 3 per cent. of old people needing permanent institutional care would be nearer the mark for Bristol, due no doubt to the disrupting effect of seafaring and urbanization on family life in this city. The housing shortage, aggravated by the bombing during the war, has also worsened the position.

The condition in which the aged sick are treated varies from one of these institutions to the other, but they all have certain features in common. There are no laboratory or X-ray departments for diagnosis on the spot; no surgical theatres or departments of special therapy such as one finds in modern general hospitals. There is a shortage of nurses which is most acute in the peripheral hospitals. Medical staffing is inadequate and, as a consequence, the standards of diagnosis and treatment were generally low. In the absence of modern methods of treatment the number of bedfast cases was, as might be expected, very high, and this made excessive demands on the inadequate nursing staff.

A geriatric centre has been established at Stapleton Hospital. Stapleton was chosen as the most promising centre for a geriatric scheme, although it was realized that the task ahead is going to be difficult. The buildings are old—they were used to house prisoners during the Napoleonic wars. The present population includes psychotics, certified and uncertified; mental defectives, ascertained and unascertained; social misfits, sexual perverts, epileptics, blind people and acute and chronic aged sick and ambulant old people.

A geriatric unit consisting of forty male and eighty female beds for active treatment has been established and a physiotherapy department with three physiotherapists and modern equipment is already functioning. When the young people are discharged there will be room for special departments for X-rays, a laboratory and a small operating theatre: and also long stay annexes for senile dementia cases, frail ambulants and ambulants awaiting discharge. The hospital will house a total of about 700 patients. Special wards for congestive cardiac failure cases have been found necessary and are functioning already. Later there will be special wards for nutritional and metabolic diseases and for orthopaedic cases. This will ensure contact with other branches of medicine and prevent the tendency to isolate the aged sick.

On the administrative side it is proposed to regulate the admissions for the whole clinical area from Stapleton, allocating to each hospital in the geriatric group the cases for which it is best fitted. An almoner's department will deal with admissions and discharges. The problem of discharging convalescents—particularly frail ambulants—is already acute. We believe that if we could dispose of the numbers already accumulated in these two categories the number of aged sick on waiting lists for admission to hospital would shrink to an insignificant figure. Unfortunately, the disposal of these old people is the subject of infinite haggling between the Hospital and Health Authorities throughout the country and we must await a decision by Parliament for the solution of this very serious problem.

### REFERENCES

Rowntree, B. S. (1947).—*Old People: Report of a Survey Committee on the problems of ageing and the care of old people.* Nuffield Foundation, Oxford University Press.

Sheldon, J. H. (1948).—*The Social Medicine of Old Age.* Nuffield Foundation, Oxford University Press.

